# Recent Development of Polydopamine Anti-Bacterial Nanomaterials

**DOI:** 10.3390/ijms23137278

**Published:** 2022-06-30

**Authors:** Zhengwei Xu, Tingting Wang, Junqiu Liu

**Affiliations:** 1 Key Laboratory of Organosilicon Chemistry and Material Technology, Ministry of Education, College of Material, Chemistry and Chemical Engineering, Hangzhou Normal University, Hangzhou 311121, China; zhengweixuhznu@163.com; 2 Department of Biomedical Engineering, College of Design and Engineering, National University of Singapore, Singapore 117583, Singapore

**Keywords:** polydopamine, adhesion, modification, polydopamine-based composites, antibacterial

## Abstract

Polydopamine (PDA), as a mussel-inspired material, exhibits numerous favorable performance characteristics, such as a simple preparation process, prominent photothermal transfer efficiency, excellent biocompatibility, outstanding drug binding ability, and strong adhesive properties, showing great potential in the biomedical field. The rapid development of this field in the past few years has engendered substantial progress in PDA antibacterial materials. This review presents recent advances in PDA-based antimicrobial materials, including the preparation methods and antibacterial mechanisms of free-standing PDA materials and PDA-based composite materials. Furthermore, the urgent challenges and future research opportunities for PDA antibacterial materials are discussed.

## 1. Introduction

Bacteria, as tiny ancient creatures, have a huge impact on human daily life. Except for some beneficial probiotics, most bacteria result in infectious diseases, which are one of the most serious health problems in the world, threatening human life [1,2,3]. The development of antibiotics has been one of the major advances in medical science over the last century, and since penicillin was first discovered, antibiotics have been a top priority in treating bacterial infections [4]. However, bacteria rapidly develop drug resistance with the widespread use of antibiotics [5]. They have evolved target modification mechanisms, inactivating enzymes and osmotic barriers to evade the attack of antibiotics [6,7]. In addition, antibiotics cause great damage to the nervous system and liver, and have severe impacts on eyesight and hearing, which are extremely unhealthy [8,9]. Therefore, many other antibacterial agents, from natural biomacromolecules, such as antibacterial enzymes [10,11] and antibacterial peptides [12,13] to synthetic compounds or polymers, such as quaternary ammonium compounds [14,15], metal ions [16] and graphene oxide sheets [17], have been gradually developed and have shown highly effective antibacterial ability. These antibacterial drugs can be divided into the contact antibacterial type and the release antibacterial type, according to antibacterial strategies. Nevertheless, each of the above antibacterial agents has some drawbacks. For instance, antibacterial enzymes or antibacterial peptides are highly sensitive to proteolysis and are therefore unstable in vivo. Synthetic polycations have poor biocompatibility, complicated synthesis/purification processes, and may also cause drug resistance after long-term therapy [18]. The uncontrolled release and severe cytotoxicity of metal ions limit their wide application [19]. Moreover, these antibacterial agents also have other disadvantages such as a high cost of raw materials, potential biological risks and possible environmental pollution. For the above reasons, it is particularly important to develop new antibacterial materials that are safe, effective and environmentally friendly. At the same time, the preparation process of the materials need to be as simple and convenient as possible in order to facilitate the promotion of the materials.

Polydopamine (PDA), an artificial melanin-like biopolymer, is a mussel-inspired material from mussel adhesion proteins, which has excellent adhesion to almost all types of substrates. Since the emergence of mussel-inspired PDA, it has attracted more and more interest due to its simple preparation procedure, outstanding biocompatibility and low cytotoxicity, and it has been extensively utilized in the fields of biomaterials, energy and catalysts [20,21,22,23,24]. It is worth noting that the excellent performances of PDA in terms of photothermal conversion, ultraviolet shielding, electrochemistry and biocompatibility are mainly attributed to the hierarchical physical and chemical performances [25]. Specifically, the active catechol groups and primary amine groups on PDA endow it with outstanding adherence and metal coordination [26,27]. The catechol groups can form covalent bonds with amino- or thiol-terminal reagents through different chemical reactions, which facilitates the grafting of small molecules, biomolecules and polymers to PDA surfaces [28,29]. Furthermore, catechol can also act as a building block for biomaterials through metal chelation effects, hydrogen bonding, electrostatic interactions, etc., providing efficient modification sites [30,31,32]. Based on the inherent qualities of PDA, it has gradually emerged as a promising candidate for antibacterial materials. On the one hand, it has a superior photothermal conversion effect and it adheres to almost all types of surfaces. On the other hand, it provides a simple and universal method for functionalizing material surfaces to gain various multifunctional materials. To date, metal ions and antibacterial drugs can be connected with PDA substances to build an antibacterial interface [33,34,35,36,37]. In conclusion, PDA-based materials are a superior choice for building multifunctional antimicrobial platforms.

**Scheme 1:**
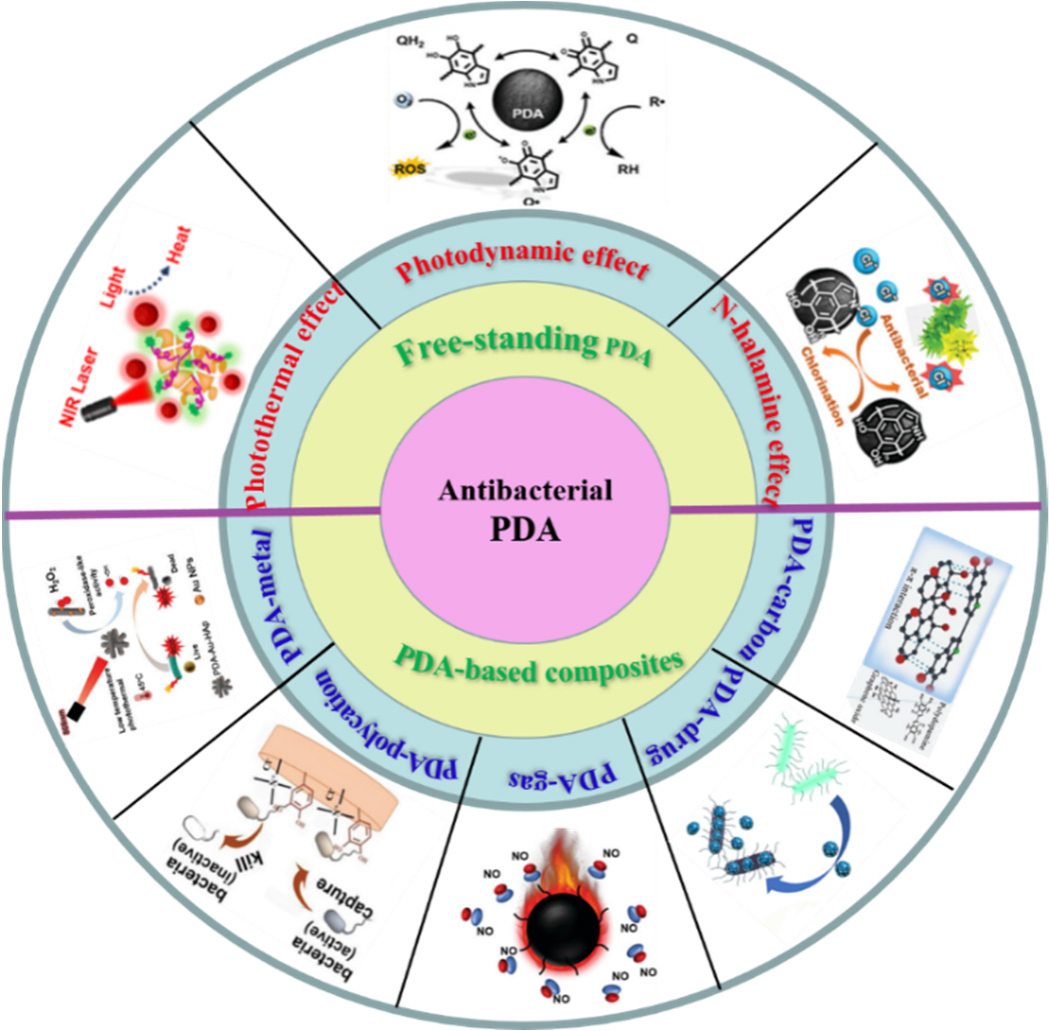
The antibacterial mechanisms based on PDA antibacterial material.

Research on the modification of PDA to various materials is progressing rapidly, and the goal of this review is to determine antibacterial mechanisms based on PDA materials (Scheme 1). First, we describe the preparation and antibacterial mechanism of PDA as a free-standing antibacterial material. After that, we present extensive research on the excellent antibacterial properties of PDA-based composites, including PDA-metal ions, quaternary ammonium salts (QAS), nitric oxide (NO), antibacterial drugs, carbon nanotubes and graphene oxide (GO). At the end, we summarize and prospect the challenges and future research directions of PDA-based antibacterial materials.

## 2. Free-Standing PDA Anti-Bacteria

PDA has become a promising antibacterial material because of its unique antimicrobial properties. To date, there have been numerous studies on the antimicrobial mechanism of PDA. Basically, the main antibacterial mechanisms of PDA can be classified into the following three types: (1) Photothermal antibacterial therapy—PDA has a broad range of light absorption from ultraviolet to near-infrared wavelengths, and it has an outstanding photothermal conversion effect [38]; (2) photodynamic antibacterial therapy—due to the abundant active catechol groups in PDA, ROS can be generated to destroy bacterial cell walls [39]; (3) N-halamine antibacterial therapy—PDA can be chemically modified to N-halamine by halogenation reaction, which can provide efficient PDA antibacterial performance through the destructive effect of halide ions [40].

### 2.1. Photothermal Antibacterial Therapy

In recent years, photothermal therapy (PTT) based on photothermal conversion materials has been widely used in the field of antibacterials. It is a non-invasive method of inducing local hyperthermia using light irradiation, which destroys the integrity of pathogenic bacteria [41]. Compared with some traditional classical antibacterial methods, PTT has a broad spectrum of antibacterial activity without bacterial resistance [42,43]. The antibacterial mechanism of the photothermal effect is to utilize enough heat to destroy the cell membrane of microorganisms, resulting in the leakage of the contents, which is a non-invasive therapeutic effect through the cell thermal ablation pathway. Therefore, PTT, as a novel heating way with excellent biocompatibility and high selectivity, is considered to be a safe, efficient and environmentally friendly strategy for the therapy of bacterial infections [44,45]. These desirable properties of PTT have attracted considerable attention by opening up the possibility of utilizing non-invasive treatment technologies to fight bacterial infections and avoid drug resistance. The photosensitizer plays the most important role in the sterilization system of PTT. It is of great significance to explore new photosensitizers with superior photothermal conversion properties, especially with biocompatible and environmentally friendly antibacterial materials [46].

**Figure 1:**
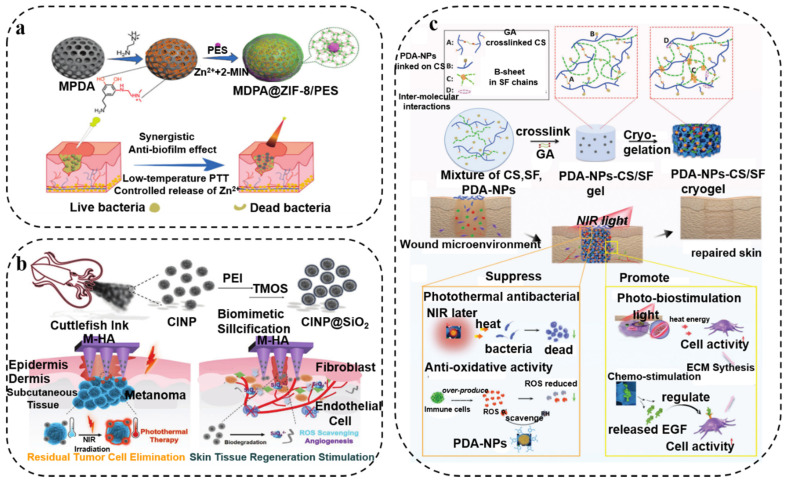
(**a**) Schematic of imidazole framework (ZIF-8)-coated mesoporous polydopamine (MPDA) core–shell nanoparticle-loaded Pifithrin-μ (PES). Reprinted with permission from [50] (copyright: The Royal Society of Chemistry). (**b**) Schematic representation of biomineralized melanin nanoparticles by SiO_2_ (CINP@SiO_2_). Reprinted with permission from [51] (copyright: Wiley-VCH GmbH, 2022). (**c**) Schematics of mussel-inspired PDA-NP-chitosan/silk fibroin (PDA-NPs-CS/SF) cryogels. Reprinted with permission from [52] (copyright: The Royal Society of Chemistry, 2019).

PDA-based photothermal nanomaterials have shown a widespread range of biomedical applications in killing bacteria [47,48,49]. PDA-based antibacterial therapy has recently been recognized as a very feasible and effective method for eradicating bacterial biofilm infections. Recently, Peng et al. [50] investigated the loading of zeolite-based imidazole framework (ZIF-8) consisting of zinc ions and 2-methylimidazole on the surface of mesoporous PDA to form a core–shell structure with polydopamine as the core and imidazole framework as the shell. The near-infrared light excitation of the core–shell nanostructures can effectively destroy bacterial biofilms, thereby realizing a low-temperature PTT (~45 °C) nanosystem with beneficial antibacterial properties (Figure 1a). Subsequently, Yu and coworkers [51] utilized natural melanin nanoparticles extracted from cuttlefish ink (CINP) with antioxidant and photothermal functions to achieve PTT. Furthermore, CINPs were encapsulated within an amorphous silica shell as a source of bioactive SiO_4_^4−^ to stimulate skin tissue regeneration. Because of the physical penetration properties of the microneedles, the CINP@SiO_2_-HA MNs gained can achieve photothermal inhibition of *S. aureus* infection (Figure 1b). In addition, combining fluorescence resonance energy transfer analysis and the photothermal properties of PDA, a smart nanoprobe with fluorescence imaging and antibacterial properties can be constructed. For example, Shen’s group [52] established a stimuli-responsive intelligent nanoprobe for efficient *S. aureus* fluorescence monitoring and near-infrared photothermal activity on-demand sterilization through the outstanding photothermal ability of PDA. PDA can also be directly doped into the hydrogel as a photothermal agent. Lu and co-workers [53] prepared a mussel chitosan/silk fibroin cryogel with near-infrared light-responsive PDA-NPs, which can be used as a multifunctional platform to adjust the wound microenvironment and promote effective wound healing (Figure 1c). Furthermore, this cryogel exhibited photothermal-assisted antibacterial activity to prevent bacterial invasion.

### 2.2. Photodynamic Antibacterial Therapy

**Figure 2:**
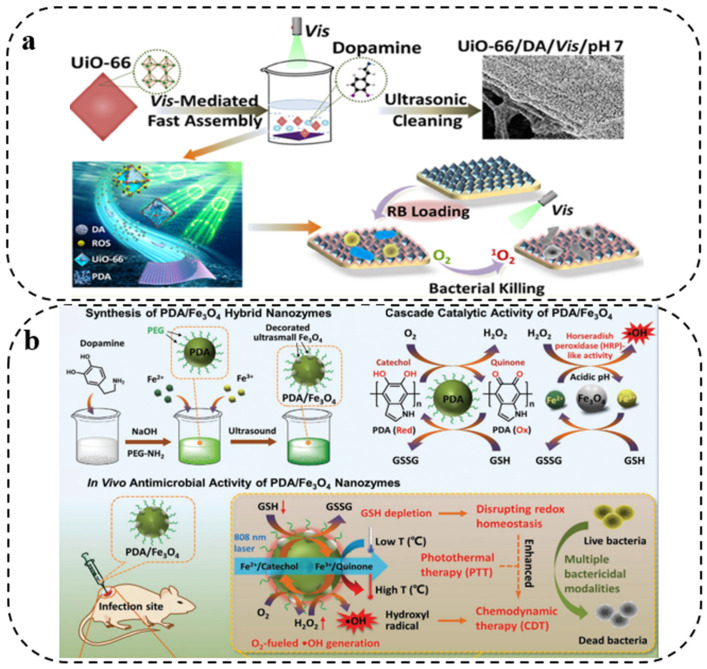
(**a**) Schematic illustration of surface assembling of UiO-66 onto substrates Reprinted with permission from [57] (copyright: American Chemical Society, 2020). (**b**) Schematic representation Synthesis of PDA/Fe_3_O_4_ hybrid nanozymes. Reprinted with permission from [59] (copyright: Wiley-VCH, 2022).

Due to the overuse of antibiotics, the problem of bacterial resistance has become increasingly serious in recent years, resulting in an increasing mortality rate from infectious diseases. Therefore, there is an urgent need to seek new drugs and new means that can effectively alleviate the problem of drug resistance. With the advancement of optical technology and the development of new photosensitizers, photodynamic therapy (PDT) has recaptured the attention of researchers because of its advantage of not easily developing drug resistance. It can effectively produce a great deal of reactive oxygen species (ROS), improve the antibacterial effect, and provide a promising method for eliminating biofilm, becoming one of the most promising treatments for drug-resistant bacterial infection [54,55]. It was reported more than a decade ago that melanin might have redox activity that could sustain ROS generation by catalyzing electron transfer from the endogenous extracellular domain to O_2_, thereby providing an additional active antimicrobial mechanism [56]. Being melanin-like, PDA can both accept electrons from reducing agents and transfer them to oxidizing agents. Liu et al. [39] used electrochemical reverse engineering to confirm that PDA can both accept electrons from reducing agents and donate electrons to various oxidants. They observed that if the electron donor is oxygen, PDA could generate ROS; if the donor was a free radical, it could quench oxidative free radicals. The electron-donating ability of PDA was determined by its redox state and greatly affected by external factors such as metal ion binding and near-infrared irradiation. The ROS generated by PDA might disrupt the bacterial cell wall and membrane, eventually resulting in bacterial death. On this basis, Hao et al. [57] prepared a stable dopamine (DA)–metal organic frameworks (MOF) composite structure under visible light irradiation by one-step mixing of DA with MOF in a neutral aqueous solution, and generated reactive oxygen, which provided excellent photodynamic antibacterial ability against Gram-positive *S. aureus* and Gram-negative *E. coli* (Figure 2a). Subsequently, Su and colleagues [58] constructed PDA–curcumin (Cur) nanocomposites based on the self-polymerization of oxidative dopamine hydrochloride and Cur under alkaline conditions, exhibiting excellent antibacterial performance against Gram-positive and Gram-negative bacteria. Furthermore, He and coworkers [59] constructed intelligent Fe_3_O_4_-modified PDA hybrid nanozymes, which can continuously convert oxygen into highly toxic hydroxyl radicals via a glutathione cascade redox reaction for chemodynamic-therapy-mediated bacterial clearance and wound disinfection (Figure 2b).

### 2.3. N-Halamine Antibacterial Therapy

As a significant halogen-based antimicrobial agent, N-halamine has obtained increasing attention for its chemical properties and practical applications. Compared with other antimicrobial agents, N-halamine is superior in many ways, including effectiveness against a broad spectrum of microorganisms, long-term physicochemical stability, and high structural durability [60,61,62,63]. Generally, N-halamine contains halogenated amides, imines, or amine groups that can act directly on thiols or amino groups in protein biological receptors, eventually causing cell inactivation [64]. Because of the abundance of amino groups in PDA, PDA can be an ideal precursor for the preparation of antibacterial N-halamine.

**Figure 3:**
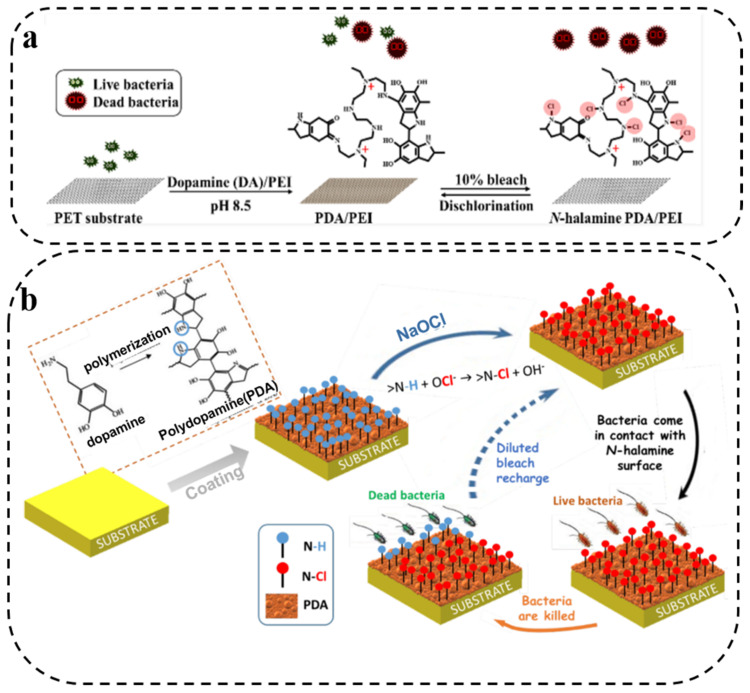
(**a**) Illustration of the chlorination of a co-deposited PDA/PEI. Reprinted with permission from [65] (copyright: American Chemical Society, 2021). (**b**) Schematic representation of the general strategy of formation and regeneration of an N-halamine coating based on polydopamine. Reprinted with permission from [66] (copyright: American Chemical Society, 2020).

Based on this, Chien et al. [65] prepared a PDA and polyethyleneimine coating by co-deposition, which could then be chlorinated. They observed better bactericidal effects against *S. aureus* and *E. coli* as the chlorine content gradually increased (Figure 3a). Similarly, Chiu and coworkers [40] also adopted this strategy, and the obtained N-halamine PDA coating exhibited not only good stability and reproducibility, but also excellent antibacterial activity. Further, Chouvy et al. [66] reported the formation of chloramine functional groups on the coating surface by soaking PDA coatings in aqueous NaOCl solution for chlorination. Compared to the unchlorinated PDA coating, the antibacterial effect of the chlorinated PDA coating was improved significantly (Figure 3b). These studies indicate that PDA can exhibit better antibacterial properties through halamine, providing a new possibility for constructing high-efficiency antibacterial materials.

## 3. PDA Composite Synergistic Antibacterial Therapy

Owing to its rich chemical properties and excellent biocompatibility, as well as its photothermal, antioxidant and adhesion capacities, PDA has seen widespread application in antibacterial research on functional composite materials [37,67]. PDA, as a surface modification material, can be combined with other antibacterial agents to produce a more efficient antibacterial effect. Here, we have compiled studies on the surface modification of metal ions, antibiotics and other antibacterial substances by PDA, which can be easily linked by chemical modification or physical adsorption to achieve controlled release, detoxification and other effects. The advantages of PDA surface modification are not only a strong interaction with the substrate as well as outstanding long-term stability in most environments, but also mild reaction conditions and non-destructivity to substrates [68]. Metal ions can be chelated with the catechol group on PDA, and the silver/gold ions chelated on the surface of PDA can be directly reduced to silver/gold nanoparticles because of the strong redox activity of PDA [35]. Antibacterial drugs can be loaded onto the surface of PDA through π-π stacking, electrostatic attraction and hydrogen bonding [69]. PDA has gained increasing attention not only for the adhesion to almost all types of surfaces, but also as it provides a simple and universal method for functionalizing material surfaces as excellent antibacterial materials.

### 3.1. PDA/Metal Composite Synergistic Antibacterial Therapy

**Figure 4:**
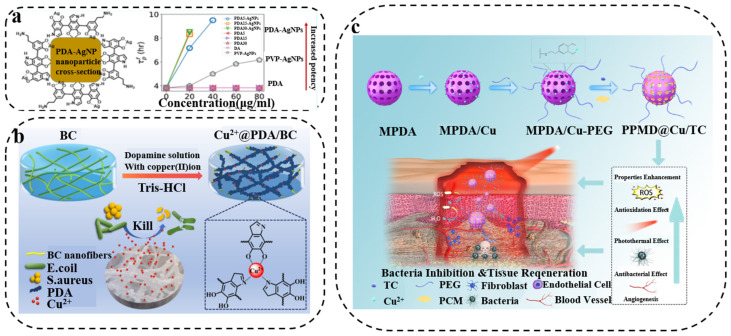
(**a**) Schematic illustration of the PDA deposition on PVP-AgNPs to form PDA-AgNPs. Reprinted with permission from [74] (copyright: American Chemical Society, 2020). (**b**) Schematic diagram of the preparation of Cu^2+^@PDA/BC composites. Reprinted with permission from [77] (copyright: Elsevier, 2021). (**c**) Schematic diagram of polydopamine nanocomposites loaded with Cu^2+^, phase change materials and tetracycline(PPMD@Cu/TC). Reprinted with permission from [78] (copyright: American Chemical Society, 2022).

Currently, metals have been developed for antibacterial applications due to their unique metallic properties. For example, Ag NPs have strong antibacterial properties due to their extremely small size, which can effectively penetrate bacterial cell membranes [70]. In addition, Ag NPs have broad-spectrum antibacterial activity without causing drug resistance, and are widely used in the development of antibacterial materials. However, when modifying Ag NPs for antibacterial applications, it is a challenge to maintain the antibacterial properties of nanoparticles, due to disadvantages such as easy aggregation. To address this issue, researchers have found that the catechol groups in PDA can reduce metal salts to metal NPs, and then the metal NPs can be fixed on a scaffold, preventing their aggregation. PDA has strong hydrophilicity because of the abundant catechol and amine groups, which makes it attract great attention as a general surface modifier [71,72]. When immobilized with Ag NPs, the surface of the PDA-modified membrane exhibits superior antibacterial performances. Besides, Ag NPs formed by the reduction of Ag ions with catechol have a more durable antibacterial effect due to their insensitivity to oxygen [73]. On this basis, Chen et al. [74] fabricated PDA-Ag NPs to study antibacterial activity. They found that the synergistic effects between the PDA coating and Ag NPs significantly enhanced the efficacy of Ag NPs against *E. coli*. This was due to the interaction of PDA with Ag that increased the production of ROS, causing significant damage to bacterial membranes (Figure 4a). Similarly, PDA-assisted Au nanomaterials can be prepared based on the large surface area, good biocompatibility and surface modification versatility of Au NPs [75].

Cu^2+^ has also attracted widespread attention due to its low preparation cost, but its toxicity at high concentrations limits further development [76]. The researchers found that the release of Cu^2+^ can be controlled by combining with PDA to achieve mild and long-lasting antibacterial ability. Based on this, Ao and coworkers [77] designed an antibacterial material based on PDA- and Cu^2+^-loaded bacterial cellulose, and the antibacterial rate of the composite material against S. aureus and *E. coli* was as high as 99.9% (Figure 4b). In addition, PDA-assisted Cu^2+^ antibacterial materials can also be an option for wound healing. Zhang’ group [78] prepared a nanocomposite material loaded with Cu^2+^ and tetracycline in mesoporous PDA NPs, which prevented the uncontrolled release of Cu^2+^ and TC and achieved the efficacy of efficient sterilization and promotion of wound healing (Figure 4c). PDA can also enhance antibacterial activity by combining with other metals. Gedanken’s group [79] prepared a PDA composite by depositing Zn-doped CuO (Zn@CuO) particles on PDA, which demonstrated excellent antibacterial ability. PDA can stabilize metal ions through chelation to maintain original performance. Most importantly, the addition of PDA not only does not increase the biotoxicity of the composites, but also greatly enhances the antibacterial properties. Therefore, PDA provides a new route for the construction of antibacterial composites.

### 3.2. PDA/Polycation Composite Synergistic Antibacterial Therapy

With the development of antibacterial agents, a large number of new cationic antibacterial agents have been widely studied and reported. Cationic antibacterial polymers can kill/inhibit the growth of microorganisms on surfaces or in the surrounding environment, change the permeability of bacterial walls, and destroy bacterial structures [80]. Cationic compounds such as quaternary ammonium, phosphonium salts, imidazolium, and pyridinium are widely used for their biocidal effect against a broad spectrum of bacteria [81,82,83]. However, their short-term antibacterial effect and leaching of potentially environmentally toxic antibacterial agents limit their further application. Since the abundant chemical performances of PDA provide lots of reaction sites, these problems can be easily solved by the connection of PDA with polycation through copolymerization, physical adsorption or chemical modification. The participation of PDA can also bring good cell/ tissue adhesion properties.

**Figure 5:**
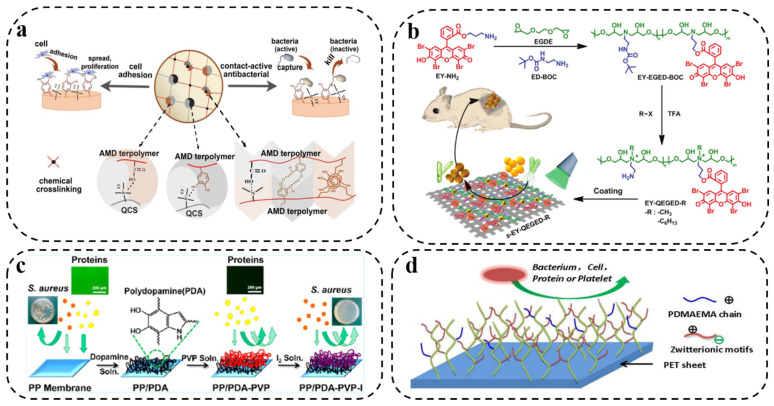
(**a**) Design strategy of cell-affinitive and contact-antibacterial AMD-QCS hydrogel. Reprinted with permission from [84] (copyright: Wiley-VCH, 2018). (**b**) Schematic illustration of the antibacterial coating formed by eosin Y (EY)-based antibacterial polycation via polydopamine. Reprinted with permission from [86] (copyright: Wiley-VCH, 2018). (**c**) Scheme of coating polydopamine (PDA) on a PP membrane as well as subsequent PVP and iodine complexation. Reprinted with permission from [68] (copyright: American Chemical Society, 2013). (**d**) Schematic diagram of surface-activated ATRP on PET sheet surface. Reprinted with permission from [87] (copyright: Elsevier, 2016).

Inspired by this, Lu et al. [84] prepared a contact-active antibacterial hydrogel by copolymerizing methacrylamide dopamine (MADA) with 2-(dimethylamino)ethyl methacrylate and quaternized chitosan to form an interpenetrating network. The reactive catechol group of MADA endowed the hydrogel with contact-enhanced bactericidal activity, as it increased the contact of bacterial cells with the positively charged groups of the hydrogel, enhancing bactericidal ability (Figure 5a). This contact-active antimicrobial hydrogel was reported to be a prospective material for wound healing, with the dual functions of facilitating tissue regeneration and avoiding bacterial infection. Besides, Li et al. [85] constructed an injectable self-healing hydrogel with antibacterial and antifouling properties via the self-assembly of a triblock copolymer containing catechol and QAS. For the physical adsorption of PDA coating, Zhu and coworkers [86] firstly combined the photosensitizer eosin Y (EY) with QAS to fabricate a novel cationic polymer based on the ring-opening reaction. The polymer was then coupled to the PDA-coated surface, resulting in a multifunctional system with synergistic antibacterial and antifouling abilities under light irradiation (Figure 5b). Moreover, the polycation can also be grafted onto the PDA coating by chemical modification. For example, Xu and coworkers [68] firstly coated a hydrophobic polypropylene (PP) porous membrane with a PDA layer, and then modified it through multiple hydrogen bonds between poly(N-vinyl pyrrolidone) (PVP) and PDA to develop an antifouling and antibacterial polymer membrane (Figure 5c). Shen’s group [87] combined bromoalkyl initiators with PDA-coated PET sheets and then used the ATRP reaction to polymerize polycarboxybetaines and polysulfobetaines in situ (Figure 5d). The amphoteric polymer formed on the surface of the PET sheet exhibited excellent blood compatibility and anti-biofouling properties. PDA/polycationic composite materials provide an effective method for antibacterial and antifouling effects, and the addition of PDA not only promotes the biocompatibility of antibacterial materials, but also brings excellent cell/tissue adhesion properties.

### 3.3. PDA/Gas Composite Synergistic Antibacterial Therapy

**Figure 6:**
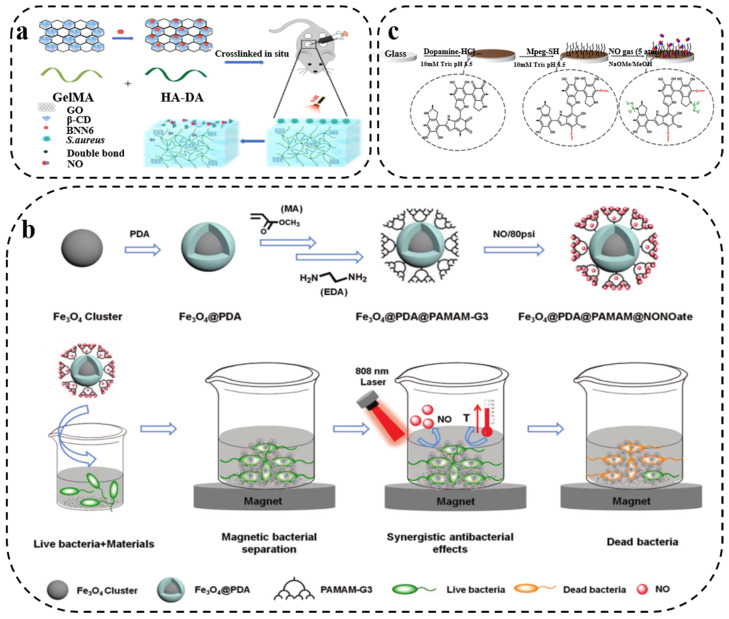
(**a**) Schematic illustration of functionalized GO nanovehicles with nitric oxide (NO) release and photothermal activity-based hydrogels (GelMA/HA-DA /GO-βCD-BNN6) [93] (copyright: American Chemical Society, 2020). (**b**) Schematic illustration of NO-releasing dendritic Fe_3_O_4_@PDA@PAMAM@NONOate nanocomposites, synergistic photothermal and NO killing of bacteria. Reprinted with permission from [94] (copyright: Wiley-VCH, 2018). (**c**) Preparation of PDA, PDA-PEG, and PDA-PEG-NO coatings. Reprinted with permission from [95] (copyright: American Chemical Society, 2019).

Gas therapy, such as with nitric oxide (NO), hydrogen (H_2_) or carbon monoxide (CO), is a promising novel non-antibiotic approach and has been extensively investigated because of its strong bactericidal ability and low drug-resistance risk [88,89,90]. Oxidation/nitrification of NO can lead to lipid peroxidation, bacterial membrane rupture, DNA cleavage and protein dysfunction, and has become one of the most widely explored broad-spectrum antibacterial gases in antimicrobial applications [91,92]. PDA has prominent biocompatibility and low biotoxicity, and can be modified on diverse implanted surfaces. It can achieve combined antibacterial effects with NO, showing outstanding application prospects in combating bacterial drug resistance. For example, Deng and coworkers [93] reported a photothermal activity-based drug consisting of NO donor BNN6 grafting onto β-cyclodextrin (β-CD)-functionalized GO and intercalating them into methacrylate-modified gelatin (GelMA)/hyaluronic acid grafted dopamine (HA-DA) hydrogels. Under near-infrared laser, GO-β-CD-BNN6 can produce hyperthermia via the photothermal effect of GO, and the nanocarrier can release NO to exert photothermally derived synergistic antibacterial efficacy (Figure 6a). The synergistic effect of photothermal and gas therapy is expected to enhance antimicrobial ability and reduce drug resistance. Similarly, Xue et al. [94] firstly used PDA-coated iron oxide (Fe_3_O_4_@PDA) as a photoconversion agent, and then grafted third-generation dendritic poly(amidoamine) (PAMAM-G3) onto the surface of Fe_3_O_4_@PDA, followed by NO loading to form NONOate to obtain multifunctional Fe_3_O_4_@PDA@PAMAM@NONOates nanocomposites. The composite can simultaneously trigger hyperthermia and NO release under laser irradiation, thereby realizing synergistic photothermal and NO antibacterial effects (Figure 6b). In addition, antibacterial interfaces with NO-releasing ability have also been reported. Boyer’s group [95] designed an antibacterial film with low fouling and NO release capability, which was covered on a glass substrate due to the multipurpose adhesion properties of PDA, followed by Michael addition to graft poly(ethylene glycol) (Figure 6c). The coating released NO for more than 48 h, effectively killing Gram-negative *P. aeruginosa* and Gram-positive *S. aureus*. In PDA/NO composite materials, PDA can maintain a high NO load with on-demand gas release, and achieve synergistic antibacterial effects with the photothermal effect, which provides a new strategy for the preparation of bacteriostatic agents with more efficient antibacterial activity.

### 3.4. PDA/Antibacterial Drug Composite Synergistic Antibacterial Therapy

**Figure 7:**
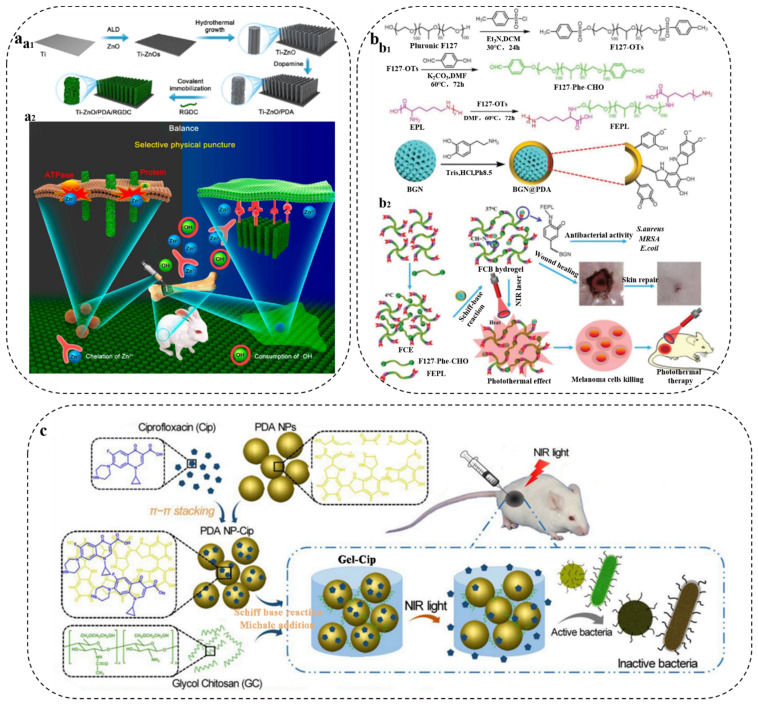
(**a**) Schematic illustration of the fabrication process of the hybrid ZnO/PDA/RGDC NR arrays on Ti (**a1**) and balancing bacteria-osteoblast competition on Ti modified with ZnO/PDA/RGDC hybrid NR arrays in vivo (**a2**). Reprinted with permission from [97] (copyright: American Chemical Society, 2017). (**b**) Schematic illustration of the preparation (**b1**) and application (**b2**) of the nanocomposite hydrogel in tumor therapy and wound healing. Reprinted with permission from [99] (copyright: Wiley-VCH, 2021). (**c**) Schematic illustration of the Gel-Cip synthetic route and antibacterial property of on-demand antibiotic release triggered by NIR light irradiation. Reprinted with permission from [102] (copyright: Elsevier, 2019).

At present, antibacterial drugs can be classified into two categories according to antibacterial methods. One type consists of contact antibacterial drugs. Most of the antibacterial drugs in contact are cations, which generate the rupture of bacterial cell membranes and kill bacteria through electrostatic action, e.g., peptides [94,96]. Because of the strong adhesive property and abundant active groups of PDA, PDA–drug conjugates can be formed by chemical modification or physical adsorption to achieve better synergistic antibacterial effects. For instance, Wu and coworkers [97] designed and fabricated a hybrid ZnO/PDA/arginine-glycine-aspartic acid-cysteine (RGDC) nanorod array, and RGDC peptide molecules could be covalently grafted onto PDA by Michael addition or Schiff base reaction (Figure 7a1). The nanorod array not only effectively killed bacteria based on contact antibacterial drugs, but also enhanced the ability to induce osteogenesis (Figure 7a2). Similarly, Fu’s group [98] also adopted this strategy as they modified PDA with an antimicrobial peptide, magainin I, which can interact with bacteria and cause significant bacterial death under near-infrared irradiation. In addition, Lei et al. [99] developed a multifunctional bioactive nanocomposite hydrogel with self-healing and antibacterial capabilities based on a bioactive polypeptide (Figure 7b1). The multifunctional hydrogel was prepared by incorporating PDA-functionalized bioactive glass nanoparticles into F127-ε-poly-L-lysine hydrogel, which not only exhibited superior self-healing and injectable properties, but also robust antibacterial ability, especially against multidrug-resistant bacteria associated with skin tumors and wound healing (Figure 7b2).

Furthermore, releasing antibacterial drugs is another antibacterial method. The commonly used antibacterial drugs such as antibiotics have broad application [100,101], but the abuse of antibiotics has generated the increase in bacterial resistance, and the biological toxicity of some antibiotics cannot be ignored. Therefore, in order to make the use of antibiotics more efficient and safer, researchers have linked antibiotics with other ingredients for long-term, on-demand release of the drug. PDA can be used as an anchoring agent for antibiotics, and antibiotics can be loaded on PDA by chemical modification or physical adsorption to prepare antibacterial materials with long circulation. For example, Wu’s group [102] prepared an injectable hydrogel by mixing ciprofloxacin-loaded PDA with glycol chitosan. As seen from Figure 7c, under near-infrared (NIR) laser irradiation, the hyperthermia generated by PDA had the dual effect of accelerating ciprofloxacin release and causing bacterial thermal lysis. Moreover, Lee and coworkers [103] developed a novel hybrid titanium (Ti) with excellent biocompatibility and antimicrobial effects by immobilizing ceftazidime (CFT) onto Ti substrates coated by PDA/polyethyleneimine using a chemical reaction. Because of the cleavage of the labile amide bond, CFT can be rapidly released under acidic conditions to achieve antibacterial ability against both Gram-positive and Gram-negative bacteria. Similarly, Ran et al. [104], Ma et al. [105], and Xu et al. [106] modified PDA with different antibiotic compounds, and then released the antibiotic to effectively inhibit or kill bacteria. PDA has played an important role in PDA/antibacterial drug composites, by, e.g., prolonging circulation time, promoting on-demand drug release, and increasing drug loading.

### 3.5. PDA/Carbon Composite Synergistic Antibacterial Therapy

Researchers have developed a new generation of antibacterial agents that rely on carbon nanomaterials (CNMs), including zero-dimensional carbon dots, one-dimensional carbon nanotubes (CNT), and two-dimensional graphene. Owing to their unique physicochemical properties and structural characteristics, CNMs have high antibacterial activity and are considered as ideal antibacterial nanomaterials to replace antibiotics [107,108]. However, because of their inert surface and poor biocompatibility, their wide application in the field of antibacterials is still limited, and PDA can modify the surface of carbon materials by its strong adhesion ability and abundant active groups to construct carbon composite antibacterial materials with better antibacterial ability.

**Figure 8:**
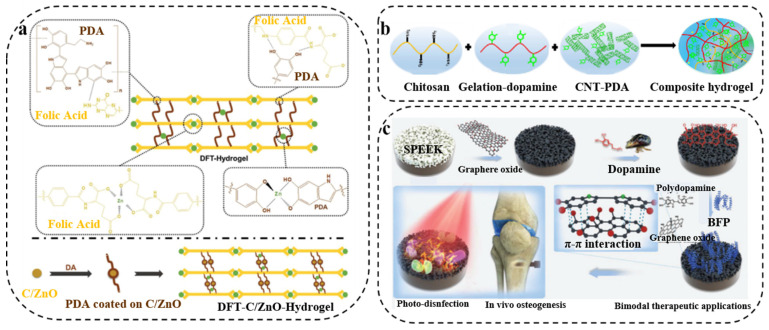
(**a**) Schematic of PDA-grafted nanoparticles to functionalize hydrogel. Reprinted with permission from [110] (copyright: Wiley-VCH, 2019). (**b**) Schematic representation of PDA-coated CNT-based composite hydrogels. Reprinted with permission from [111] (copyright: Elsevier, 2019). (**c**) Schematic illustration of the photo-disinfection and osteogenic abilities of PDA/graphene oxide/oligopeptide-decorated SPEEK implant. Reprinted with permission from [112] (copyright: Royal Society of Chemistry, 2020).

PDA/carbon composite materials are widely used in antibacterial research owing to their outstanding biocompatibility and photothermal conversion efficiency. Gedanken et al. [109] fabricated a PDA/nitrogen-doped carbon dots composite with antimicrobial property against both Gram-negative and Gram-positive bacteria. Wu and coworkers [110] also developed an antibacterial composite material based on PDA and carbon dots. They first prepared an injectable hydrogel, and then PDA-encapsulated carbon quantum dot-modified ZnO nanoparticles in the hydrogel, which exhibited efficient antibacterial effects because of the excellent photodynamic and photothermal properties provided by PDA and carbon dots (Figure 8a). In addition, Guo’s group [111] fabricated a PDA/chitosan/CNT composite hydrogel, and the PDA/CNT endowed the hydrogel with outstanding photothermal effect and broad-spectrum photothermal antimicrobial activity, giving it excellent antibacterial capacities against Gram-positive *S. aureus* and Gram-negative *E. coli*, as well as improved dispersibility of CNT (Figure 8b).

Furthermore, with the introduction of PDA, CNMs can be combined with antibacterial drugs such as metal ions to obtain more efficient synergistic antibacterial ability. For example, Tang et al. [113] constructed a composite material of Ag-deposited PDA coating on graphene nanosheets, which demonstrated prominent antibacterial ability against both Gram-negative and Gram-positive bacteria owing to the synergistic effect of graphene and Ag nanoparticles. Kundu and coworkers [114] obtained a PDA/reduced graphene oxide and Ag NP-doped a bacteria-cellulose composite membrane based on a green and environmentally friendly preparation method, which was considered to be a potential antibacterial material. Besides, Chen et al. [112] developed a multifunctional nanocoating to assemble graphene oxide, PDA and oligopeptide onto the surface of porous sulfonated polyetheretherketone (Figure 8c). The composite coating films not only significantly facilitated in vivo osseointegration and bone remodeling, but was also able to produce a powerful antibacterial phototherapy effect.

## 4. Conclusions and Perspectives

In recent years, there has been considerable and increasing interest in the study of antibacterial PDA, facilitating the investigate and development of PDA-based materials. PDA has several advantages, including great biocompatibility, strong adhesion, excellent photothermal effects as well as drug loading capacity, and negligible toxicity, providing new ways to explore and create advanced antibacterial materials. In this review, we reported the current advances and development prospects of PDA-based antibacterial materials. PDA as a novel antibacterial agent has gradually appeared in the field of biomedicine. Since PDA is rich in catechol, amine, imine and other active groups, it can be combined with many other antibacterial substances such as Ag NPs, QAS, NO, antibiotics and CNMs through chemical coupling or physical adsorption to achieve better synergistic antibacterial ability. The PDA composite antibacterial material not only has efficient antibacterial ability and superior biocompatibility, but also realizes the controlled release of antibacterial substances, which makes PDA one of the most promising materials in the field of antibacterial and implantation interfaces.

Although significant advances in PDA-based antimicrobial materials have been made, there are still some challenges to overcome. How to effectively utilize this material is the main challenge so far. Despite the use of well-defined monomeric precursors, the structure of PDA is still elusive, and the comprehension of the polymerization mechanism of PDA remains controversial. A further investigation of the internal structure and polymerization of PDA can have a direct impact on the optimization of PDA functional modification with a view to achieving better antibacterial effects. Furthermore, when dealing with applications of PDA in biomedical and related fields, some key issues should be considered, such as the long-term stability and toxicity of PDA during its ability to remain in organisms. As a crucial structural and functional component of antibacterial composites, PDA has broad research prospects and great application values in the future. We hope this review can provide ideas and insights for biomedical researchers interested in developing novel antimicrobial materials and techniques, as well as for clinicians and healthcare providers seeking alternative therapies for drug-resistant bacteria.

## Data Availability

Not applicable.
